# Reduced susceptibility of western corn rootworm (*Diabrotica virgifera virgifera* LeConte) populations to Cry34/35Ab1-expressing maize in northeast Nebraska

**DOI:** 10.1038/s41598-022-23755-z

**Published:** 2022-11-10

**Authors:** Jordan D. Reinders, Lance J. Meinke

**Affiliations:** Department of Entomology, University of Nebraska, Lincoln, NE 68583 USA

**Keywords:** Entomology, Plant sciences

## Abstract

The western corn rootworm (WCR; *Diabrotica virgifera virgifera* LeConte) is a significant pest of maize (*Zea mays* L.) across the United States Corn Belt. Transgenic maize hybrids expressing insecticidal proteins derived from *Bacillus thuringiensis* (Bt) have been used to manage WCR since 2003. Widespread resistance to Cry3Bb1 (and associated cross-resistance to mCry3A and eCry3.1Ab) has placed increased selection pressure on Cry34/35Ab1 in single-protein and pyramided transgenic maize hybrids. Data on the susceptibility of Nebraska WCR populations to Cry34/35Ab1 has not been published since 2015 and plant-based bioassays conducted in 2017–2018 confirmed resistance to Cry3Bb1 + Cry34/35Ab1 maize, suggesting resistance to Cry34/35Ab1 has evolved in the Nebraska landscape. Therefore, plant-based bioassays were conducted on F_1_ progeny of WCR populations collected from northeast Nebraska in 2018 and 2019. Larval survival and development were used to classify resistance to Cry34/35Ab1 in each WCR population. Bioassays confirmed incomplete resistance to Cry34/35Ab1 maize in 21 of 30 WCR populations; 9 of 30 WCR populations remained susceptible to Cry34/35Ab1. Collectively, results indicate that northeast Nebraska WCR populations were in the initial stages of resistance evolution to Cry34/35Ab1 during 2018–2019. Appropriate resistance management strategies are needed to mitigate resistance and preserve efficacy of rootworm-active products containing Cry34/35Ab1.

## Introduction

The transgenic era for management of the western corn rootworm (WCR; *Diabrotica virgifera virgifera* LeConte) began in the early 2000s after commercialization of maize (*Zea mays* L.) expressing insecticidal proteins derived from *Bacillus thuringiensis* (Bt) Berliner. Maize hybrids expressing single-protein Cry3Bb1^1^, Cry34/35Ab1 (now reclassified as Gpp34Ab1/Tpp35Ab1^2^)^3^, or mCry3A^4^ were widely adopted among growers in the United States (US) Corn Belt^5^ because of excellent maize root protection and handling/application of soil-applied insecticides was eliminated^6^. Transgenic plant technologies eventually replaced soil and foliar insecticides as the primary tactics used in continuous maize production (i.e., ≥ 2 consecutive growing seasons of cultivation to manage corn rootworms)^7^. However, none of the commercially available Bt proteins targeting the WCR are expressed at high-dose levels^8–13^ (i.e., 25× dose necessary to kill 99% of susceptible insects^14^) and repeated use of individual Bt proteins in maize hybrids has facilitated WCR field-evolved resistance to these proteins in areas of the US Corn Belt^15–22^. Cross-resistance among the Cry3 proteins (e.g., Cry3Bb1, mCry3A, eCry3.1Ab) has been observed^15,16,23,24^, although differences in protein structure inhibit the development of cross-resistance between the three-domain Cry proteins and the Cry34/35Ab1 binary protein, creating two independent mechanisms of toxicity against WCR^24,25^.

Cultivation of pyramided transgenic maize hybrids that contain two or more modes of action targeting an individual pest have increased in recent years to mitigate resistance evolution^5^. Rootworm-active pyramids registered in the US include Cry3Bb1 + Cry34/35Ab1^26^, mCry3A + Cry34/35Ab1^27–29^, mCry3A + eCry3.1Ab^30^, and Cry3Bb1 + Cry34/35Ab1 + DvSnf7 dsRNA^31^. The widespread reports of field-evolved resistance to Cry3Bb1 throughout the US Corn Belt and cross-resistance among the Cry3 proteins has increased selection pressure on the Cry34/35Ab1 component of many rootworm-active pyramids. In 2013, Gassmann et al.^20^ collected six WCR populations from Iowa maize fields with significant root damage to single-protein or pyramided Bt maize hybrids containing Cry34/35Ab1. Single-plant bioassays indicated incomplete resistance (i.e., decreased survival and/or development on Bt versus non-rootworm Bt maize but higher survival on Bt maize versus susceptible colonies^32^) to Cry34/35Ab1 in WCR populations collected from fields with significant larval feeding damage to single-protein Cry34/35Ab1 maize and maize pyramided with Cry34/35Ab1 and a Cry3 protein^20^. This was the first confirmation of WCR field-evolved resistance to Cry34/35Ab1 maize in the US Corn Belt. During the same year, a WCR population was collected from a Minnesota maize field planted to Cry3Bb1 + Cry34/35Ab1 with > 1 node of injury per plant and larval bioassays confirmed incomplete resistance to Cry34/35Ab1 in single-plant and diet-based bioassays^18^.

Single-plant larval bioassays conducted on Iowa WCR populations collected in 2017 showed increased mean proportional and corrected survival on Cry34/35Ab1-expressing maize relative to 2013 bioassays, suggesting the magnitude of resistance to Cry34/35Ab1 had increased during that time span^21^. Complete resistance (i.e., no difference in bioassay survival or development between Bt and non-rootworm Bt maize) was observed in one WCR population^21^, which contrasts with previous studies only documenting incomplete resistance to Cry34/35Ab1 in WCR^18,20^. In October 2018, Corteva Agriscience informed the US Environmental Protection Agency that Cry34/35Ab1 resistance was confirmed using diet-based bioassays in WCR populations in Delaware County, Iowa^33^. A high frequency of WCR resistance to Cry34/35Ab1 was also confirmed in Illinois populations collected in 2020, with no significant difference in single-plant bioassay survival between Cry34/35Ab1 maize and non-rootworm Bt maize observed in some populations^34^.

In northeast and southwest Nebraska, WCR populations collected in 2011 and 2012 exhibited low mean proportional and corrected survival on Cry34/35Ab1 maize that was not significantly different from susceptible laboratory colonies in single-plant bioassays^16^. Further plant-based bioassays conducted on WCR populations collected in 2013 and 2014 indicated that WCR susceptibility to Cry34/35Ab1 maize was maintained in the geographical areas previously bioassayed^35^. Single-plant bioassays of WCR populations collected from maize fields in northeast Nebraska in 2017 and 2018 confirmed resistance to the Cry3Bb1 + Cry34/35Ab1 pyramid^22^. Resistance to the Cry3Bb1 + Cry34/35Ab1 pyramid was incomplete in most WCR populations; however, complete resistance was observed in two WCR populations^22^. The presence of complete resistance to the Cry3Bb1 + Cry34/35Ab1 pyramid suggests that some level of resistance to Cry34/35Ab1 is present in the Nebraska landscape due to the additive function of each trait within the pyramid^15,36,37^. Previous studies have confirmed widespread resistance to Cry3 proteins in Nebraska, with an increasing frequency of populations exhibiting complete resistance over time^16,22,35,38,39^. Understanding the current susceptibility of Nebraska WCR populations to Cry34/35Ab1 is necessary to refine integrated pest management (IPM) and insect resistance management (IRM) strategies when deploying transgenic technologies that include Cry34/35Ab1.

Therefore, this study was conducted to characterize the variability in susceptibility of WCR populations collected from northeast Nebraska to Cry34/35Ab1 maize. Single-plant larval bioassays^15^ were conducted on F_1_ progeny from 30 WCR populations collected from continuous maize fields in northeast Nebraska in 2018 and 2019 and compared to susceptible WCR colonies. This area of the state was chosen due to the high volume of continuous maize production (three to > 10 consecutive years) to provide feed for the confined livestock industry and the associated long-term use of single-protein or pyramided maize hybrids containing Cry34/35Ab1 to manage potential WCR injury. Mean WCR head capsule width and fresh weight were measured to identify the impact of sublethal exposure to Cry34/35Ab1 maize on larval development. Bioassay results will provide updated data on the susceptibility of Nebraska WCR populations to Cry34/35Ab1 maize and inform development of resistance management recommendations.

## Methods

### Western corn rootworm populations

**Figure 1:**
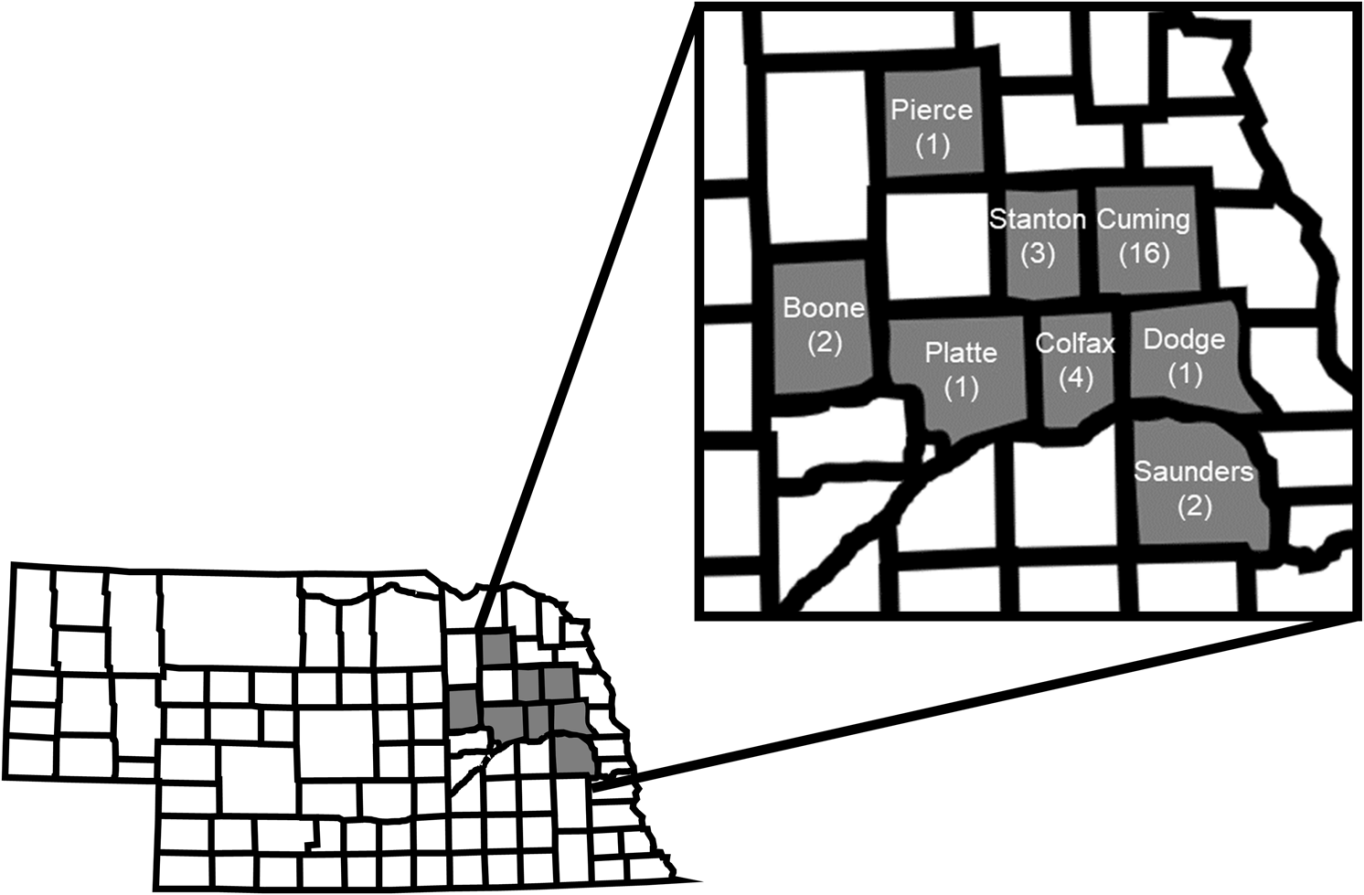
Nebraska state map showing counties from which western corn rootworm populations were collected in gray. The number of western corn rootworm populations collected from each county is indicated in parentheses.

A minimum of 50 gravid WCR females were collected from 30 commercial maize fields across eight northeast Nebraska counties during the fall of 2018 (17–30 August) and 2019 (31 July–28 August; Fig. 1). The counties from which WCR populations were collected included Boone (2 populations), Cuming (16 populations), Colfax (4 populations), Dodge (1 population), Pierce (1 population), Platte (1 population), Saunders (2 populations), and Stanton (3 populations). Each WCR population was assigned a unique number to differentiate populations (Tables 1, 2). Laboratory colonies of diapausing WCR maintained at the USDA-ARS North Central Agricultural Research Laboratory (Brookings, SD) were used as a susceptible control (LAB-S) during each year bioassays were conducted. Initial WCR populations were collected from Butler County, Nebraska (1990), Potter County, South Dakota (1995), Finney County, Kansas (2000), and Centre County, Pennsylvania (2000) prior to the commercialization of rootworm-Bt proteins in 2003 and have been continuously reared without the addition of wild-type genes to preserve Bt susceptibility.

**Table 1:** Corrected survival (± SE) of western corn rootworm populations assayed on Cry34/35Ab1 maize during 2019.

County	Population	Cry34/35Ab1 corrected survival ± SE^a^	Classification of Cry34/35Ab1 resistance
Cuming	1	0.261 ± 0.07cd	Incomplete^b^
Cuming	2	0.211 ± 0.02d	Incomplete
Cuming	3	0.019 ± 0.01f	Susceptible^c^
Cuming	4	0.519 ± 0.09ab	Incomplete
Cuming	5	0.160 ± 0.05de	Susceptible
Cuming	6	0.190 ± 0.07de	Susceptible
Cuming	7	0.560 ± 0.12ab	Incomplete
Cuming	8	0.315 ± 0.07bcd	Incomplete
Cuming	9	0.133 ± 0.05def	Susceptible
Cuming	10	0.306 ± 0.05bcd	Incomplete
Cuming	11	0.656 ± 0.09a	Incomplete
Stanton	12	0.429 ± 0.08abc	Incomplete
Colfax	13	0.455 ± 0.06ab	Incomplete
Saunders	14	0.086 ± 0.04ef	Susceptible
Susceptible laboratory control	LAB-S	0.065 ± 0.01ef	X

^a^Corrected survival values followed by the same lowercase are not significantly different (*p* > 0.05); ^b^incomplete resistance criteria: (1) significantly greater survival on Cry34/35Ab1 maize compared to the LAB-S control, and (2) within a population, proportional survival and/or larval development was significantly lower on Cry34/35Ab1 maize relative to non-RW Bt maize; ^c^susceptible criteria: no difference in survival on Cry34/35Ab1 maize compared to the LAB-S control.

**Table 2:** Corrected survival (± SE) of western corn rootworm populations assayed on Cry34/35Ab1 maize during 2020.

County	Population	Cry34/35Ab1 corrected survival ± SE^a^	Classification of Cry34/35Ab1 resistance
Cuming	15	0.053 ± 0.04de	Susceptible^b^
Colfax	16	0.324 ± 0.07b	Incomplete^c^
Pierce	17	0.217 ± 0.06bc	Incomplete
Stanton	18	0.264 ± 0.06bc	Incomplete
Cuming	19	0.157 ± 0.05cde	Incomplete
Boone	20	0.043 ± 0.03e	Susceptible
Cuming	21	0.265 ± 0.08bc	Incomplete
Cuming	22	0.258 ± 0.07bc	Incomplete
Cuming	23	0.400 ± 0.09b	Incomplete
Boone	24	0.351 ± 0.06b	Incomplete
Platte	25	0.253 ± 0.06bc	Incomplete
Stanton	26	0.200 ± 0.06bcd	Susceptible
Dodge	27	0.000 ± 0.00e	Susceptible
Colfax	28	0.306 ± 0.05b	Incomplete
Saunders	29	0.222 ± 0.07bc	Incomplete
Colfax	30	0.670 ± 0.05a	Incomplete
Susceptible laboratory control	LAB-S	0.046 ± 0.01e	X

^a^Corrected survival values followed by the same lowercase are not significantly different (*p* > 0.05); ^b^susceptible criteria: no difference in survival on Cry34/35Ab1 maize compared to the LAB-S control; ^c^incomplete resistance criteria: (1) significantly greater survival on Cry34/35Ab1 maize compared to the LAB-S control, and (2) within a population, proportional survival and/or larval development was significantly lower on Cry34/35Ab1 maize relative to non-RW Bt maize.

### Plant-based larval bioassays

Field-collected WCR adults were maintained by population in 28 cm^3^ plexiglass cages at the University of Nebraska-Lincoln under laboratory conditions after collection. Eggs obtained from each WCR population were placed in Petri dishes (Fisher Scientific, Waltham, MA) containing moistened soil substrate and were maintained at room temperature for 1-month post-oviposition and subsequently held at 10 °C for 1 month and 7 °C for 3–5 months to facilitate diapause development and termination^16,40^. Post-diapause development and egg hatch was facilitated by placing a subset of ~ 10,000 eggs from each WCR population at 25 °C for 14–17 days to obtain F_1_ neonate progeny for use in on-plant bioassays. The large egg sample size led to synchronous hatch of enough eggs per population to infest a complete bioassay in 1 day. Due to obligatory diapause in WCR, plant-based bioassays were conducted on F_1_ progeny during the spring/summer of the year following adult collection (i.e., 2019 and 2020).

The Gassmann single-plant larval bioassay was used in this study^15^. It is a standardized technique used to detect small shifts in WCR susceptibility to Bt proteins. For bioassays conducted in this study, two maize hybrids were used: (1) a hybrid expressing Cry34/35Ab1 and (2) its non-rootworm Bt near-isoline (hereafter ‘non-RW Bt’). Twelve plants of each hybrid were grown in individual 1L plastic pots (Johnson Paper & Supply Co., Minneapolis, MN) until the V4-V5 growth stage^41^ to assay each WCR population. Twelve randomly selected F_1_ neonate larvae were then placed on the roots of each individual plant and pots were held at 24 °C with a 14:10 (L:D) photoperiod for 17 days. Individual plants and surrounding soil were then placed in a Berlese funnel (40 W, 120 V lightbulbs) for 4 days to extract larval survivors.

Larval survivors from each plant were placed on a Kimwipe (Kimberly-Clark Worldwide, Inc., Roswell, GA) for 3 min to absorb excess ethyl alcohol prior to measuring larval development metrics. Head capsule width of larval survivors was measured using an AmScope 3.5 –90× Simul-Focal Trinocular Stereo Zoom microscope with attached 18MP USB3 Camera (United Scope LLC, Irvine, CA) to the nearest 0.01 μm. Fresh weight was measured by weighing larval survivors from each maize plant on an OHAUS Voyager PRO VP413CN precision balance (OHAUS Corporation, Pine Brook, NJ) to the nearest 1 mg. Both larval head capsule width and fresh weight increase as larvae develop through each of three instars^42^. Therefore, comparison of Bt versus non-RW Bt larval development metrics provides an indirect characterization of larval development after sublethal exposure to Bt proteins^21,38,43^.

### Statistical analysis

#### Proportional survival

Proportional survival on each maize plant was calculated by dividing the number of larval survivors by 12. Survival data from 2019 and 2020 were analyzed separately. Initial analyses indicated a similar response among the four susceptible WCR colonies to Cry34/35Ab1 maize in 2019 (*F*_3,44_ = 1.15; *p* = 0.3382) and 2020 (*F*_3,44_ = 1.98; *p* = 0.1306) bioassays. Therefore, proportional survival data from all individual susceptible WCR colonies was pooled within a bioassay year to create a composite sample. A generalized linear mixed model (GLMM; GLIMMIX procedure^44^) following a binomial distribution with a logit link function^45,46^ was used to evaluate proportional larval survival on each maize hybrid. Fixed factors included WCR population, maize hybrid, and their interaction. Plant observation nested within the interaction of WCR population and maize hybrid was included in the model as a random factor to control for an overdispersion of variance^46^. The SLICE statement was used to identify significant differences in proportional survival between maize hybrids within each WCR population. Tukey’s multiplicity adjustment was used to control for type I error rates when making pairwise comparisons. Dunnett’s adjustment was used to make multiplicity comparisons of proportional survival within the Cry34/35Ab1 hybrid of each WCR field population relative to the LAB-S control.

#### Larval development metrics

The SQL procedure^44^ was used to average head capsule width for larval survivors on each bioassay plant. Mean fresh weight of larval survivors was calculated on a per-plant basis by dividing the total weight of all larvae from an individual plant by the number of larval survivors on the same plant. Analyses for head capsule width and fresh weight were conducted separately and by bioassay year. A linear model [GLIMMIX procedure^44^] was used to calculate the mean head capsule width or fresh weight of larval survivors per maize hybrid for each WCR population. WCR population, maize hybrid, and their interaction were included in the model as fixed factors. Normality assumptions and model fit were evaluated by examining residual plots. Significant differences in larval development between maize hybrids within each WCR population and differences between field populations and the LAB-S control were identified using the same statistical procedures outlined previously for proportional survival.

#### Classification of resistance to Cry34/35Ab1 maize

Based on proportional survival and larval development data, each WCR population was classified as susceptible, incompletely resistant, or completely resistant to Cry34/35Ab1 maize. WCR field populations that did not exhibit significantly higher proportional survival on Cry34/35Ab1 maize relative to the LAB-S control were classified as susceptible. The following criteria were used to classify WCR field populations as incompletely resistant: (1) proportional survival on Cry34/35Ab1 maize was significantly greater than the LAB-S control, and (2) proportional survival on Cry34/35Ab1 maize was significantly lower than survival on non-RW Bt maize, or (3) larval development was significantly decreased on Cry34/35Ab1 maize compared to non-RW Bt maize. WCR field populations were classified as completely resistant if: (1) proportional survival on Cry34/35Ab1 maize was significantly greater than the LAB-S control, and (2) no differences in proportional survival or larval development were observed between Cry34/35Ab1 and non-RW Bt maize hybrids.

#### Corrected survival on Cry34/35Ab1 maize

Corrected survival on Cry34/35Ab1 maize was calculated for each WCR population as the complement of corrected mortality using Abbott’s correction^47^ by dividing survival on each Cry34/35Ab1-expressing maize plant by mean survival of the WCR population on the non-RW Bt hybrid^21,22^. A linear model [GLIMMIX procedure^44^] following a normal distribution with unequal variances between populations was used to evaluate corrected survival on Cry34/35Ab1 maize. WCR population was included in the model as a fixed factor. Normality assumptions and model fit were evaluated by examining residual plots and heterogenous variance between populations was allowed to control for nonconstant variance by including a random statement (GROUP = population). The DIFFS option was used to identify significant differences in corrected survival on Cry34/35Ab1 maize among WCR populations.

## Results

### Single-plant larval bioassays

#### Proportional survival

**Figure 2:**
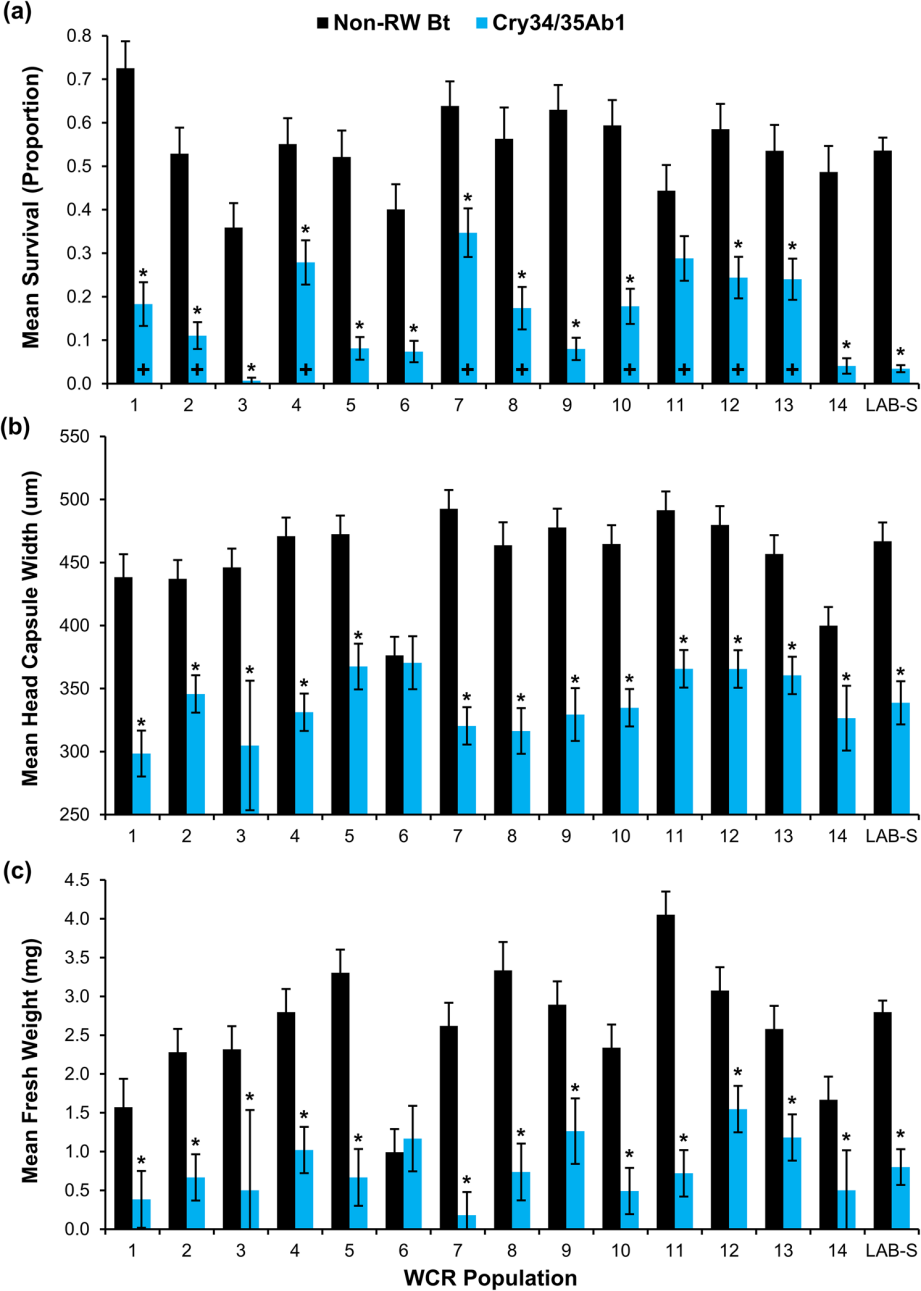
Larval survival and development of western corn rootworm populations bioassayed on non-rootworm Bt and Cry34/35Ab1 maize in 2019. (**a**) Mean proportional survival (± SE), (**b**) mean head capsule width (± SE), and (**c**) mean fresh weight (± SE). Asterisks above Cry34/35Ab1 bars indicate significantly lower survival or development compared to the non-rootworm Bt hybrid within a population (Tukey’s multiplicity adjustment, *p* < 0.05). A ‘+’ within Cry34/35Ab1 bars indicates significantly greater survival or development on Cry34/35Ab1 maize relative to the susceptible laboratory control (Dunnett’s test, *p* < 0.05).

**Figure 3:**
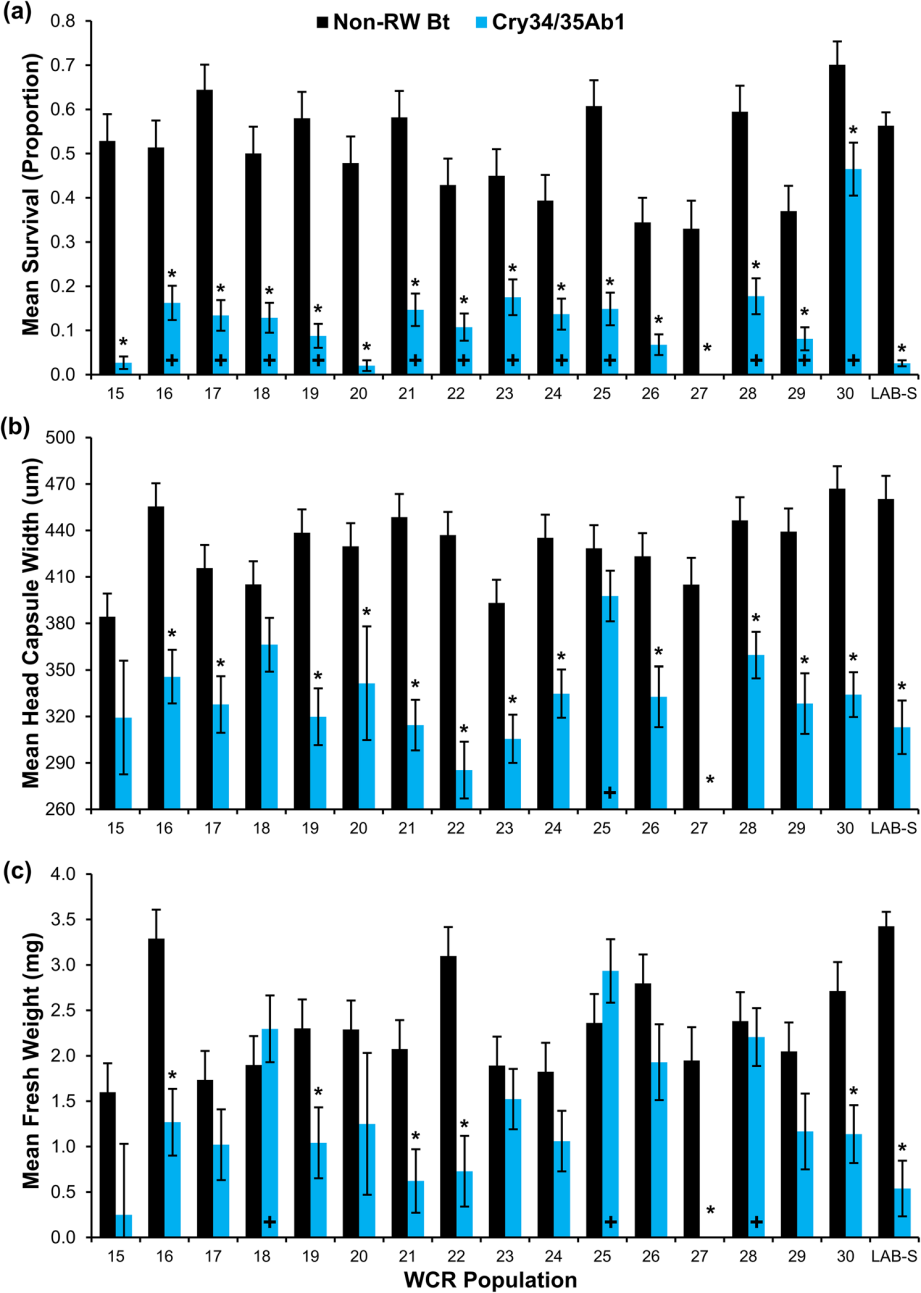
Larval survival and development of western corn rootworm populations bioassayed on non-rootworm Bt and Cry34/35Ab1 maize in 2020. (**a**) Mean proportional survival (± SE), (**b**) mean head capsule width (± SE), and (**c**) mean fresh weight (± SE). Asterisks above Cry34/35Ab1 bars indicate significantly lower survival or development compared to the non-rootworm Bt hybrid within a population (Tukey’s multiplicity adjustment, *p* < 0.05). A ‘+’ within Cry34/35Ab1 bars indicates significantly greater survival or development on Cry34/35Ab1 maize relative to the susceptible laboratory control (Dunnett’s test, *p* < 0.05).

The interaction between WCR population and maize hybrid significantly influenced mean WCR proportional survival in 2019 (*F*_14,386_ = 5.70; *p* < 0.0001) and 2020 (*F*_15,432_ = 4.76; *p* < 0.0001) bioassays. A significant decrease in survivorship on Cry34/35Ab1 maize relative to non-RW Bt maize was observed in 13 of 14 WCR field populations assayed in 2019 (Fig. 2a) and all 16 WCR field populations assayed in 2020 (Fig. 3a). In both 2019 and 2020 bioassays, proportional survival of the LAB-S control was significantly lower on Cry34/35Ab1 maize compared to non-RW Bt maize. Relative to the LAB-S control, significantly higher survival on Cry34/35Ab1 maize was observed in nine of 14 WCR field populations in 2019 bioassays (Fig. 2a) and 12 of 16 WCR field populations in 2020 bioassays (Fig. 3a).

#### Larval development metrics

The interaction between WCR population and maize hybrid significantly influenced mean WCR larval survivor head capsule width in 2019 (*F*_14,276_ = 2.60; *p* = 0.0015) and 2020 (*F*_15,303_ = 2.40; *p* = 0.0027) bioassays. A significant decrease in mean larval survivor head capsule width on Cry34/35Ab1 maize relative to non-RW Bt maize was observed in 13 of 14 WCR field populations assayed in 2019 (Fig. 2b) and 13 of 16 WCR field populations assayed in 2020 (Fig. 3b). The mean head capsule width of LAB-S control larvae surviving exposure to Cry34/35Ab1 maize was significantly lower than survivors on non-RW Bt maize in both bioassay years (Figs. 2b, 3b). In 2019 bioassays, mean head capsule width of each WCR field population was not significantly different than LAB-S control larval survivors exposed to Cry34/35Ab1 maize (Fig. 2b). A similar trend was observed in 2020 bioassays, except for WCR population 25 which had a significantly greater mean larval head capsule width among Cry34/35Ab1 survivors than recorded for LAB-S control survivors (Fig. 3b). When visualized as proportion of larvae in specific instars, larval survivors from WCR field populations exposed to Cry34/35Ab1 maize reached predominately second instar (2018: 0.699 ± 0.06; 2019: 0.695 ± 0.04) while many larval survivors on non-RW Bt maize reached third instar (2018: 0.810 ± 0.05; 2019: 0.776 ± 0.04; Supplementary Tables S1, S2). A similar trend was observed with LAB-S control larvae surviving Cry34/35Ab1 exposure predominately reaching second instar (2018: 0.756 ± 0.11; 2019: 0.760 ± 0.11) while many LAB-S control larval survivors on non-RW Bt maize reached third instar (2018: 0.857 ± 0.12; 2019: 0.986 ± 0.01; Supplementary Tables S1, S2).

 The interaction between WCR population and maize hybrid also significantly influenced mean WCR larval survivor fresh weight in 2019 (*F*_14,323_ = 2.85; *p* = 0.0005) and 2020 (*F*_15,343_ = 4.58; *p* < 0.0001) bioassays. A significant decrease in mean larval survivor fresh weight on Cry34/35Ab1 maize relative to non-RW Bt maize was observed in 13 of 14 WCR field populations assayed in 2019 (Fig. 2c) and five of 16 WCR field populations assayed in 2020 (Fig. 3c). The mean fresh weight of LAB-S control larvae surviving exposure to Cry34/35Ab1 maize was significantly lower than survivors on non-RW Bt maize in both bioassay years (Figs. 2c, 3c). In 2019 bioassays, mean fresh weight of all WCR field populations was not significantly different than the mean of LAB-S control larval survivors (Fig. 2c). In 2020 Cry34/35Ab1 bioassays, 3 of 16 WCR field populations exhibited significantly greater mean larval fresh weight relative to LAB-S control survivors (Fig. 3c).

#### Classification of resistance to Cry34/35Ab1 maize

In 2019 bioassays, nine of 14 WCR field populations exhibited incomplete resistance to Cry34/35Ab1 maize (Table 1). A similar trend was observed in 2020 bioassays, with 12 of 16 WCR field populations exhibiting incomplete resistance to Cry34/35Ab1 maize (Table 2). The remaining field populations in each bioassay year were classified as susceptible to Cry34/35Ab1 maize. No WCR field populations met the criteria to be classified as completely resistant to Cry34/35Ab1.

#### Corrected survival on Cry34/35Ab1 maize

Corrected survival on Cry34/35Ab1 maize was significantly influenced by WCR population in both 2019 (*F*_14,46.3_ = 12.89; *p* < 0.0001) and 2020 (*F*_15,53.48_ = 15.36; *p* < 0.0001) bioassays. Significant variation in corrected survival on Cry34/35Ab1 maize was observed among WCR populations, with values ranging from 0.019 to 0.656 in 2019 bioassays (Table 1) and from 0.000 to 0.670 in 2020 bioassays (Table 2). In 2019 bioassays, WCR population 3 had the lowest numerical corrected survival (0.019), and the corrected survival of five WCR field populations was not significantly different than the LAB-S control corrected survival (Table 1). In 2020 bioassays, WCR population 27 had the lowest numerical corrected survival (0.000; no survival on Cry34/35Ab1 maize), and the corrected survival of four WCR field populations was not significantly different than the LAB-S control corrected survival (Table 2).

## Discussion

This study provides the first confirmation of a level of WCR field-evolved resistance to Cry34/35Ab1 maize in Nebraska with plant-based bioassays. WCR resistance to Cry34/35Ab1 was classified as incomplete in 70% (21 of 30) of populations assayed while the remaining 30% (9 of 30) remained susceptible to Cry34/35Ab1 (Tables 1, 2). The significant variation in proportional (Figs. 2a, 3a) and corrected survival (Tables 1, 2) among WCR populations exposed to Cry34/35Ab1 maize in bioassays indicates that a mosaic of susceptibility to this binary Bt protein is present across the northeast Nebraska landscape. Although the WCR management histories of many fields included in this study were not available, variability in agronomic and rootworm management practices at the field and farm-levels undoubtedly contributed to the mosaic of WCR susceptibility observed. The number of consecutive years in continuous maize was variable in the study area, which can influence the annual WCR density present^48^. Additionally, the history of Bt maize use (historical choice of specific single or pyramided Bt hybrids and duration of specific protein use) can significantly influence the rate of resistance evolution^49^. Of note is WCR population 27, which was collected from a site that had never been planted to maize hybrids expressing rootworm-active Bt proteins. Zero survival obtained in plant-based bioassays of this WCR population is a good indicator of how susceptible WCR populations were when Cry34/35Ab1 was first commercialized and provides additional evidence documenting the reduction of efficacy in the landscape reported in this paper. The presence of WCR populations collected in 2018 and 2019 exhibiting increased survival on Cry34/35Ab1 maize, but only low proportional and corrected survival in this study, suggests that WCR resistance to Cry34/35Ab1 maize had evolved recently during the period since previous plant-based bioassays were conducted^16,35^.

Larval development metrics from this study help confirm the relative recency of WCR resistance evolution to Cry34/35Ab1. Larval head capsule width and fresh weight can be used as surrogate criteria to differentiate the effects of sublethal exposure to Bt proteins on larval development within a WCR population. Previous research with populations susceptible to Cry34/35Ab1 has documented that mean WCR adult emergence from Cry34/35Ab1 maize can be significantly later when compared to non-RW Bt maize^50,51^, suggesting that larval development is slower on maize containing Cry34/35Ab1. Similar adult emergence patterns have also been observed in WCR populations susceptible to Cry3Bb1^51–53^. In populations highly resistant to Cry34/35Ab1, this developmental delay is less apparent or can completely disappear^21^. This was demonstrated with laboratory bioassays in several studies where survivors from WCR populations highly resistant to Cry34/35Ab1 maize had a high proportion of WCR larvae developing to third instar, similar to the proportion recovered from non-RW Bt maize^21,54^. In contrast, WCR populations exhibiting incomplete resistance were primarily in second instar compared to 50–75% of larval survivors recovered from non-RW Bt maize progressing to third instar^20^. These examples collectively suggest the inverse relationship between larval development and corrected survival observed for Cry3Bb1^43^ may also occur with Cry34/35Ab1. Developmental differences observed in this study when populations were reared on Cry34/35Ab1 and non-RW Bt maize (Figs. 2b,c, 3b,c; Supplementary Tables S1,S2) parallel the previous example when only low levels of resistance were present^20^. In addition, mean larval head capsule width and mean fresh weight of ≥ 90% of WCR field populations assayed were not significantly different from the LAB-S control (Figs. 2a–c, 3a–c). These results collectively suggest that larval development was significantly inhibited by sublethal exposure to Cry34/35Ab1 in single-plant bioassays and, in most cases, larval development rate in WCR field populations was similar to susceptible laboratory WCR colonies.

WCR populations 3 and 5 from this study were also included in F_1_ generation bioassays with Cry3Bb1 and Cry3Bb1 + Cry34/35Ab1 in a previous study (designated as C5 and C9, respectively; [reported in^22^]). These populations from the same field collections were all bioassayed in 2019 and enable insight into the additive nature of Bt traits in pyramids. Bioassays conducted on WCR populations C5 (2019) and C9 (2019) showed high corrected survival on Cry3Bb1 maize (0.864 and 0.750, respectively) and lower corrected survival on Cry3Bb1 + Cry34/35Ab1 maize (0.205 and 0.271, respectively)^22^. These populations were both classified as completely resistant to Cry3Bb1 and incompletely resistant to Cry3Bb1 + Cry34/35Ab1 maize based on bioassay proportional survival and larval development metrics. In the current study, these populations were both identified as susceptible to Cry34/35Ab1 by statistical analysis of bioassay data and exhibited low corrected survival (0.019 and 0.160, respectively; Table 1). Despite the high level of resistance to Cry3Bb1 observed in these two WCR populations, the large decrease in corrected survival on Cry3Bb1 + Cry34/35Ab1 maize observed by Reinders et al.^22^ can likely be attributed to the relatively high susceptibility of these populations to Cry34/35Ab1 maize observed in this study.

In summary, we conclude that WCR populations from northeast Nebraska were in the early stages of resistance evolution to Cry34/35Ab1 maize when adult collections were made in 2018–2019. Variability in WCR susceptibility to Cry34/35Ab1 was evident throughout the landscape based on survival in plant-based bioassays, with the majority of field populations classified as incompletely resistant to Cry34/35Ab1 maize. The mosaic of WCR populations susceptible to Cry34/35Ab1 or exhibiting a low level of resistance to Cry34/35Ab1 in this study is in contrast to the common occurrence of complete resistance of WCR to Cry3Bb1 temporally reported before or during the time period of this study^22,38^. This reinforces the importance of Cry34/35Ab1 as a key component of many commercially available rootworm-Bt pyramids that contain a Cry3 protein and Cry34/35Ab1. Results of this study provide documentation of the change in susceptibility of WCR to Cry34/35Ab1 over time in Nebraska and will serve as a benchmark for comparison in future bioassays as selection pressure on Cry34/35Ab1 will continue in the near term in areas where continuous maize is grown. WCR resistance levels to Cry34/35Ab1 in plant-based bioassays have increased over time in Iowa^21^, so a similar pattern may occur over time in Nebraska with continued cultivation of maize hybrids expressing Cry34/35Ab1. The variability in corrected survival among WCR populations in this and previous studies^20–22,39^ highlights the importance of how individual fields are managed in relation to the rate of WCR resistance evolution to Cry34/35Ab1. Rotation of management tactics within an IPM framework in areas of WCR Bt resistance is vital to preserve the durability and efficacy of current rootworm-active Bt hybrids^5,55,56^. Increased emphasis on crop rotation as a tactic in WCR management programs is needed in the northeast Nebraska study area. Shifting production systems in more farms to a shorter maize-soybean rotation (i.e., 2–3 years of maize to 1 year soybean) could reduce WCR densities and selection pressure from Bt proteins compared to continuous Bt use in longer-term (4–10+ years) continuous maize production. Practical rotation of Bt proteins has not been possible because Cry34/35Ab1 is currently a component in most pyramids^26–29,31^ and the cross-resistance that occurs among current Cry3 proteins^15,16,23,24^. Until multiple new transgenic options are commercialized with unique modes of action to allow for trait rotation, use of existing trait combinations to manage WCR injury will continue to be a challenge in long-term continuous maize systems.

## Supplementary Information

Supplementary Tables.

## Data Availability

All data generated or analyzed during this study are included in this published article.
