# Supplementary material for: Reduced susceptibility of western corn rootworm (Diabrotica virgifera virgifera LeConte) populations to Cry34/35Ab1-expressing maize in northeast Nebraska
Source: Sci Rep. 2022 Nov 10;12:19221. doi: 10.1038/s41598-022-23755-z (PMC9649616; doi:10.1038/s41598-022-23755-z)
Supplement: Supplementary file 1 — Supplementary Tables. [file 41598_2022_23755_MOESM1_ESM.docx]

**Supplementary Tables from Reduced susceptibility of western corn rootworm (*Diabrotica virgifera virgifera* LeConte) populations to Cry34/35Ab1-expressing maize in northeast Nebraska**

**Supplementary Table S1.** Proportion of larvae by instar that survived exposure to non-RW Bt maize and Cry34/35Ab1-expressing maize in 2019 plant-based bioassays conducted on western corn rootworm populations collected in 2018.

|  | Non-RW Bt Maize | | | Cry34/35Ab1 Maize | | |
| --- | --- | --- | --- | --- | --- | --- |
| Population (2018) | First Instar | Second Instar | Third Instar | First Instar | Second Instar | Third Instar |
| 1 | 0.000 | 0.275 | 0.725 | 0.167 | 0.833 | 0.000 |
| 2 | 0.000 | 0.224 | 0.776 | 0.063 | 0.813 | 0.125 |
| 3 | 0.000 | 0.308 | 0.692 | 1.000 | 0.000 | 0.000 |
| 4 | 0.000 | 0.101 | 0.899 | 0.000 | 0.829 | 0.171 |
| 5 | 0.000 | 0.120 | 0.880 | 0.000 | 0.750 | 0.250 |
| 6 | 0.000 | 0.655 | 0.345 | 0.091 | 0.727 | 0.182 |
| 7 | 0.000 | 0.044 | 0.956 | 0.039 | 0.882 | 0.078 |
| 8 | 0.000 | 0.130 | 0.870 | 0.176 | 0.706 | 0.118 |
| 9 | 0.000 | 0.056 | 0.944 | 0.000 | 0.917 | 0.083 |
| 10 | 0.000 | 0.094 | 0.906 | 0.192 | 0.654 | 0.154 |
| 11 | 0.000 | 0.016 | 0.984 | 0.000 | 0.690 | 0.310 |
| 12 | 0.000 | 0.048 | 0.952 | 0.028 | 0.667 | 0.306 |
| 13 | 0.000 | 0.156 | 0.844 | 0.000 | 0.657 | 0.343 |
| 14 | 0.000 | 0.429 | 0.571 | 0.167 | 0.667 | 0.167 |
| Field Mean (± SE) | **0.000 (0.00)** | **0.190 (0.05)** | **0.810 (0.05)** | **0.137 (0.07)** | **0.699 (0.06)** | **0.163 (0.03)** |
|  |  |  |  |  |  |  |
| Finney Co., KS | 0.000 | 0.000 | 1.000 | 0.000 | 0.857 | 0.143 |
| Centre Co., PA | 0.000 | 0.506 | 0.494 | 0.000 | 1.000 | 0.000 |
| Butler Co., NE | 0.000 | 0.066 | 0.934 | 0.333 | 0.667 | 0.000 |
| Potter Co., SD | 0.000 | 0.000 | 1.000 | 0.000 | 0.500 | 0.500 |
| LAB-S Mean (± SE) | **0.000 (0.00)** | **0.143 (0.12)** | **0.857 (0.12)** | **0.083 (0.08)** | **0.756 (0.11)** | **0.161 (0.12)** |

**Supplementary Table S2.** Proportion of larvae by instar that survived exposure to non-RW Bt maize and Cry34/35Ab1-expressing maize in 2020 plant-based bioassays conducted on western corn rootworm populations collected in 2019.

|  | Non-RW Bt Maize | | | Cry34/35Ab1 Maize | | |
| --- | --- | --- | --- | --- | --- | --- |
| Population (2019) | First Instar | Second Instar | Third Instar | First Instar | Second Instar | Third Instar |
| 15 | 0.000 | 0.500 | 0.500 | 0.000 | 1.000 | 0.000 |
| 16 | 0.000 | 0.054 | 0.946 | 0.000 | 0.708 | 0.292 |
| 17 | 0.000 | 0.272 | 0.728 | 0.050 | 0.850 | 0.100 |
| 18 | 0.000 | 0.389 | 0.611 | 0.053 | 0.526 | 0.421 |
| 19 | 0.000 | 0.133 | 0.867 | 0.154 | 0.692 | 0.154 |
| 20 | 0.000 | 0.217 | 0.783 | 0.000 | 0.667 | 0.333 |
| 21 | 0.000 | 0.108 | 0.892 | 0.091 | 0.636 | 0.182 |
| 22 | 0.000 | 0.161 | 0.839 | 0.063 | 0.875 | 0.063 |
| 23 | 0.000 | 0.385 | 0.615 | 0.231 | 0.654 | 0.115 |
| 24 | 0.000 | 0.207 | 0.793 | 0.250 | 0.450 | 0.300 |
| 25 | 0.000 | 0.230 | 0.770 | 0.000 | 0.455 | 0.545 |
| 26 | 0.000 | 0.220 | 0.780 | 0.000 | 0.800 | 0.200 |
| 27 | 0.000 | 0.389 | 0.611 | No survival | No survival | No survival |
| 28 | 0.000 | 0.118 | 0.882 | 0.038 | 0.654 | 0.308 |
| 29 | 0.000 | 0.185 | 0.815 | 0.000 | 0.750 | 0.250 |
| 30 | 0.000 | 0.010 | 0.990 | 0.030 | 0.701 | 0.269 |
| Field Mean (± SE) | **0.000 (0.00)** | **0.224 (0.04)** | **0.776 (0.04)** | **0.064 (0.02)** | **0.695 (0.04)** | **0.235 (0.04)** |
|  |  |  |  |  |  |  |
| Finney Co., KS | 0.000 | 0.030 | 0.970 | 0.000 | 0.875 | 0.125 |
| Centre Co., PA | 0.000 | 0.012 | 0.988 | 0.333 | 0.667 | 0.000 |
| Butler Co., NE | 0.000 | 0.000 | 1.000 | 0.000 | 0.500 | 0.500 |
| Potter Co., SD | 0.000 | 0.013 | 0.987 | 0.000 | 1.000 | 0.000 |
| LAB-S Mean (± SE) | **0.000 (0.00)** | **0.014 (0.01)** | **0.986 (0.01)** | **0.083 (0.08)** | **0.760 (0.11)** | **0.156 (0.12)** |
